# Management of Unilateral Maxillary Lateral Incisor Agenesis With Canine Substitution and Mandibular Canine-Lateral Incisor Transposition: A Case Report

**DOI:** 10.7759/cureus.92740

**Published:** 2025-09-19

**Authors:** Hind Alfehaid, Khalid Abalkhail

**Affiliations:** 1 Department of Orthodontics, King Abdulaziz Medical City, Ministry of National Guard Health Affairs, Riyadh, SAU; 2 Department of Preventive Dental Science, College of Dentistry, King Saud bin Abdulaziz University for Health Sciences, Riyadh, SAU; 3 Department of Research, King Abdullah International Medical Research Center, Riyadh, SAU

**Keywords:** agenesis, canine, canine substitution, lateral incisor, transposition, unilateral

## Abstract

This clinical case report presents the orthodontic diagnosis and management of a 15-year-old female patient with two rare concurrent dental anomalies - unilateral agenesis of the maxillary left lateral incisor and complete transposition of the mandibular right canine and lateral incisor. The absence of tooth #22 was managed by canine substitution, with tooth #23 reshaped and positioned to simulate a lateral incisor, while tooth #24 was adapted to function as a canine. The mandibular transposition between teeth #42 and #43 was accepted and aligned in the transposed position, with careful biomechanical control of torque and angulation to optimize periodontal health.

A comprehensive non-extraction strategy was chosen, employing fixed orthodontic appliances in both arches, followed by Essix clear retainers and fixed lingual retention for long-term stability. The treatment plan emphasized esthetic recontouring, functional adaptation, and periodontal protection. Treatment was completed in 36 months, resulting in space closure in the upper arch, a good occlusion, improved smile esthetics, and functional guidance without the need for prosthetic replacement. This case underlines the importance of early diagnosis, conservative treatment planning, and multidisciplinary coordination in managing complex anomalies. It further highlights that canine substitution, combined with the acceptance of transposition, can provide a predictable and esthetic long-term outcome when implants or restorative approaches are contraindicated or challenging.

## Introduction

The coexistence of maxillary lateral incisor agenesis with canine substitution and mandibular tooth transposition is exceedingly uncommon. It requires a multidisciplinary approach and careful biomechanical planning to ensure optimal esthetic and functional outcomes. This study discusses the diagnosis, planning, and successful management of similar cases using a comprehensive and conservative orthodontic approach.

Tooth transposition, on the other hand, is a much rarer anomaly that poses a unique orthodontic dilemma [1-3]. It involves the positional interchange of two adjacent teeth within the dental arch. The mandibular lateral incisor and canine transposition is among the rarest types and can be complete (involving both crowns and roots) or incomplete (pseudo-transposition, where only the crowns are interchanged). The decision to correct or accept the transposition is dependent on several factors, including the stage of dental development, severity of displacement, esthetic concerns, and root position [1,3].

Tooth agenesis is one of the most common developmental dental anomalies and presents both esthetic and functional challenges in orthodontics [4]. It can occur in isolation or as part of a syndrome, and its management must be carefully tailored based on the patient’s age, skeletal pattern, and specific dental configuration. Maxillary lateral incisor agenesis, in particular, is frequently encountered [4] and has a prevalence ranging from 1.5% to 2% in the general population [5]. It significantly affects smile esthetics due to its visibility in the anterior maxillary segment.

Several treatment options are available to manage maxillary lateral incisor agenesis, including space maintenance for future implant placement, resin-bonded bridges, or orthodontic space closure with canine substitution [6,7]. The latter offers a more conservative and often stable long-term solution, especially in younger patients where implant placement is contraindicated due to ongoing growth. Canine substitution requires careful reshaping of the canine to simulate the lateral incisor and adjustment of the first premolar to serve as the canine functionally. This study aimed to present the orthodontic management of a patient with unilateral maxillary lateral incisor agenesis and mandibular canine-lateral incisor transposition, highlighting a conservative approach using canine substitution and acceptance of transposition to achieve functional and esthetic outcomes.

## Case presentation

**Figure 1:**
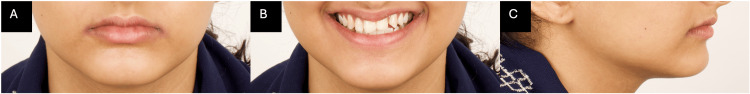
Pre-treatment extraoral photographs. The images show (A) frontal at rest, (B) frontal smiling, and (C) lateral at rest.

A 15-year-old female presented to the orthodontic department with concerns about dental crowding and esthetics. She had no significant medical history and attended regular dental checkups, maintaining good oral health. The clinical examination revealed well-balanced facial proportions and a well-aligned facial profile (Figures 1A-1C).

**Figure 2:**
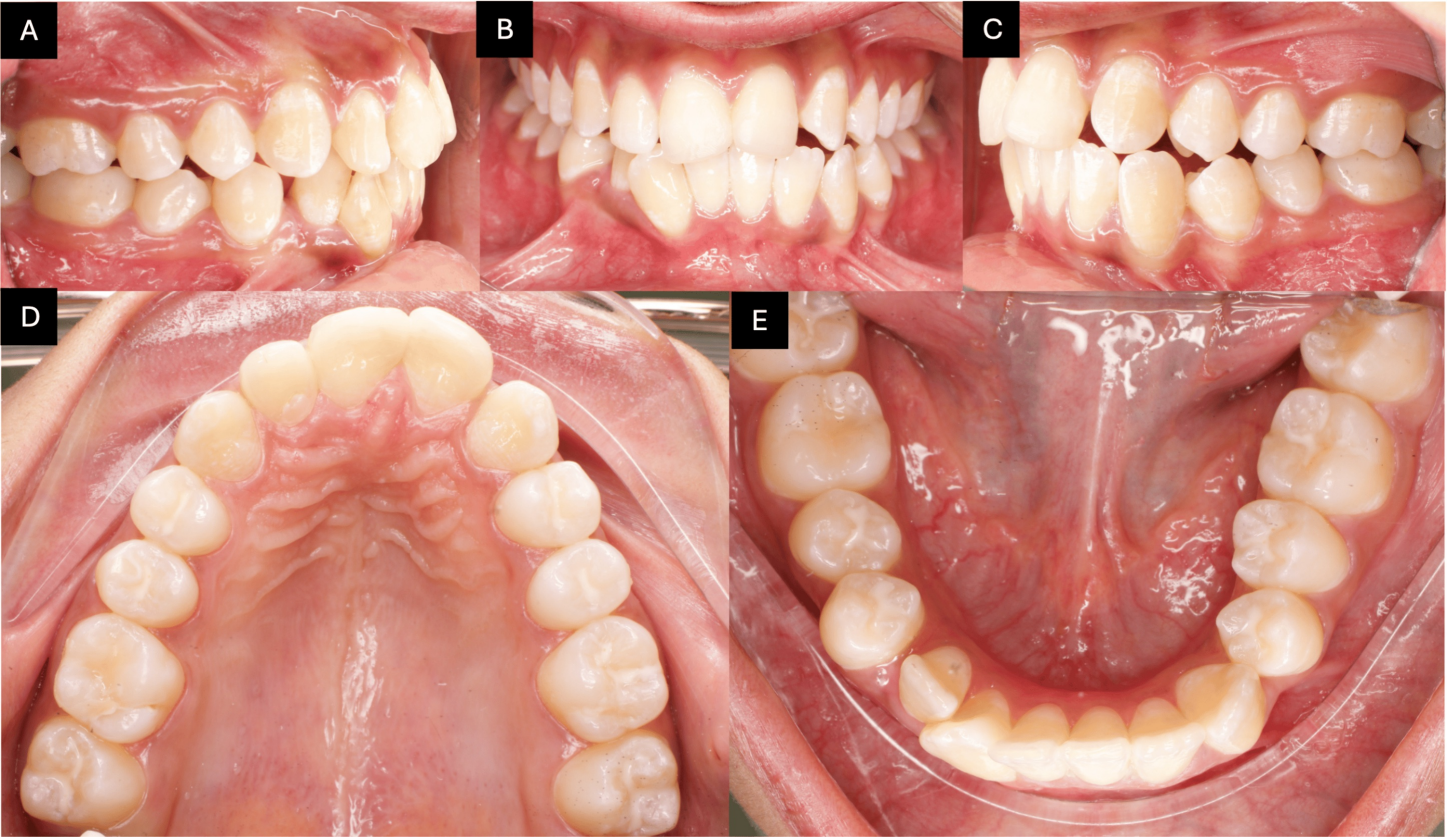
Pre-treatment intra-oral photographs. The images show (A) right buccal view, (B) frontal view, (C) left buccal view, (D) maxillary occlusal view, and (E) mandibular occlusal view.

Intra-oral examination revealed an overjet of 4 mm and an overbite of 20%, with crossbite of #23/33 and #24/34. The maxillary left lateral incisor was congenitally missing, and the upper left canine had erupted mesially into the lateral incisor position. In the mandibular right quadrant, clinical and radiographic evaluation showed a complete transposition between the right lateral incisor and canine, with both crown and root positions fully switched. On the right side, a class I molar relationship was observed, whereas on the left side, a class II molar relationship was present. Based on Angle’s classification, the case was diagnosed as class II division 1 subdivision left (Figures 2A-2E).

**Figure 3:**
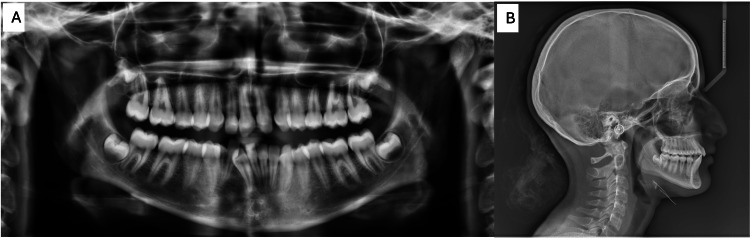
Pre-treatment radiographs. The images show (A) pre-treatment panoramic and (B) pre-treatment lateral cephalogram.

Radiographic evaluation, including panoramic imaging and a cephalogram, confirmed the agenesis of tooth #22 (upper left lateral incisor) (Figures 3A, 3B). Additionally, tooth #23 (upper left canine) was found to have erupted into the position typically occupied by tooth #22. A complete transposition was also observed between teeth #42 (lower right lateral incisor) and #43 (lower right canine).

The patient was diagnosed as class II division 1 subdivision left, unilateral agenesis of the maxillary left lateral incisor (tooth #22), mesial eruption of the maxillary left canine (tooth #23), complete transposition of the mandibular right lateral incisor and canine (teeth #42 and #43), and mild crowding in the mandibular arch, along with gingival recession related to tooth #43. The treatment objectives were to improve smile esthetics, achieve arch coordination, close spaces in the maxillary arch, relieve mandibular crowding, establish ideal overjet and overbite, and create an overall stable occlusion.

Several treatment alternatives were considered. For the maxillary arch, space opening with implant replacement of the lateral incisor was ruled out because the patient was only 15 years old and still growing, which posed a risk of infraocclusion and future bone loss around implants. A resin-bonded bridge was also excluded due to its long-term debonding risk and the patient’s preference for a natural conservative approach. For the mandibular arch, attempting correction of the transposition was considered inadvisable, as the roots were fully interchanged, making correction highly risky with respect to root resorption, periodontal loss, and extended treatment time. Consequently, the chosen treatment involved canine substitution in the maxillary arch and acceptance of the mandibular transposition, as this approach was conservative, preserved alveolar bone, avoided surgical or prosthetic intervention, and provided more esthetically stable results. Therefore, the treatment objectives focused on space closure in the maxillary arch through canine substitution, esthetic modification of tooth #23 to resemble a lateral incisor, and functional adaptation of tooth #24 to serve as a canine. Additional goals included acceptance of the mandibular transposition, comprehensive alignment and leveling of both arches, achieving a stable occlusion with functional guidance, and improving the gingival level of tooth #43 through proper alignment.

The treatment plan involved bonding fixed orthodontic appliances (McLaughlin, Bennett, and Trevisi {MBT} system 0.018-inch slot) in both arches, esthetic recontouring of tooth #23, and functional reshaping of tooth #24. The complete transposition of teeth #42 and #43 was maintained, with minor adjustments in torque and angulation. Long-term retention was planned using upper and lower clear retainers, along with fixed retainers in both arches.Both retention plans were chosen to maximize long-term stability. The fixed retainer ensured constant, invisible protection against relapse of the rotated and/or transposed anterior teeth, which are at a higher risk of positional instability. Meanwhile, the removable Essix retainer provided full-arch coverage with additional stability, particularly at night, to support long-term retention. The combination of both offered comprehensive retention, particularly for patients undergoing complex treatments, those with an increased risk of tooth movement, or those with concerns regarding patient compliance. To enable simultaneous use, the impression for the Essix retainer was taken after cementation of the fixed retainer, with instructions to the laboratory to add wax over it to create adequate space and ensure a proper fit without exerting pressure on the fixed retainer or compromising the Essix fit.

Treatment progress and outcome

Orthodontic treatment was performed using a 0.018-inch slot fixed appliance system in both arches. Initial alignment and leveling were achieved with a progressive sequence of nickel-titanium archwires, which were later transitioned to stainless steel working wires to provide control during the finishing stages. To facilitate esthetic and functional substitution, tooth #23 (the maxillary left canine, replacing the missing lateral incisor) was bonded with a lateral incisor bracket to allow for proper torque and positioning within the lateral incisor space. Concurrently, tooth #24 (the first premolar, which substitutes for a canine) was bonded with a canine bracket to ensure appropriate guidance and occlusal function in its new role.

**Figure 4:**
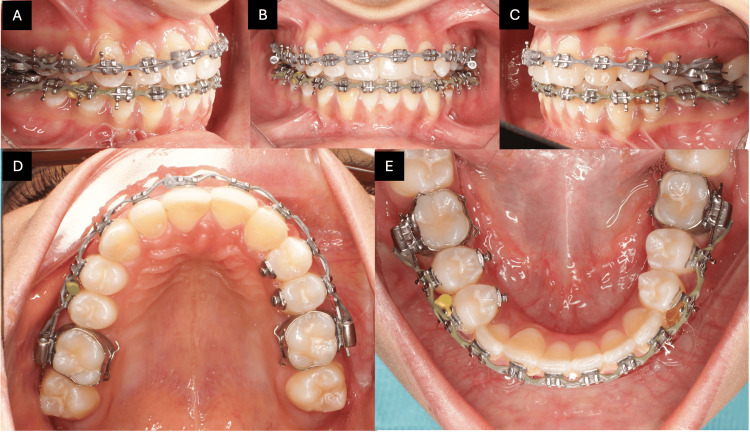
Progress intra-oral photographs. The images show (A) right buccal view, (B) frontal view, (C) left buccal view, (D) maxillary occlusal view, and (E) mandibular occlusal view.

To address the class II division 1 subdivision left and assist with midline correction and anteroposterior alignment, unilateral class II elastics were applied. On the right side, a triangular elastic configuration involving the upper canine and the lower first and second premolars was used to enhance vertical control and promote optimal intercuspation. Several teeth required derotation, which was effectively managed using rotational wedges. Additionally, power chains were employed at various treatment stages to close residual spaces. Special biomechanical attention was given to tooth #43, which was involved in a complete transposition with tooth #42. To address its buccal root prominence and improve its periodontal position within the alveolar bone, lingual root torque was incorporated into the archwire specifically for #43. This precise torque control enhanced axial inclination, improved root positioning, and supported better soft tissue adaptation, contributing to a more stable and esthetic result (Figures 4A-4E).

**Figure 5:**
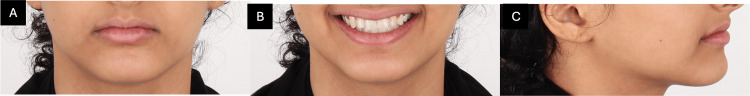
Post-treatment extraoral photographs. Images show (A) frontal at rest, (B) frontal smiling, and (C) lateral at rest.

**Figure 6:**
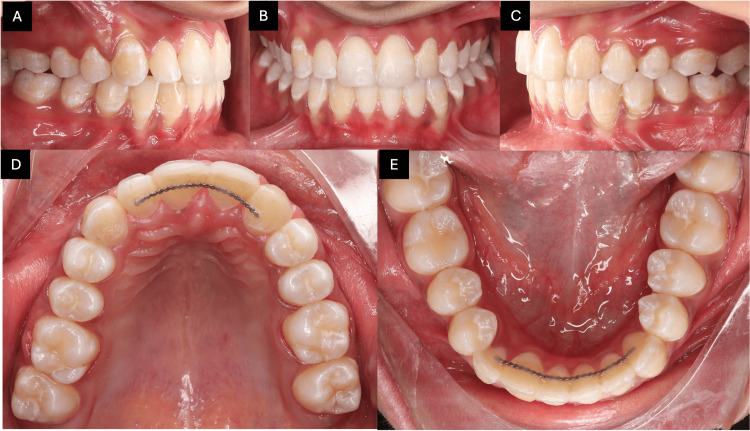
Post-treatment photographs showing final alignment and occlusion. The images show (A) right buccal view, (B) frontal view, (C) left buccal view, (D) maxillary occlusal view, and (E) mandibular occlusal view.

**Figure 7:**
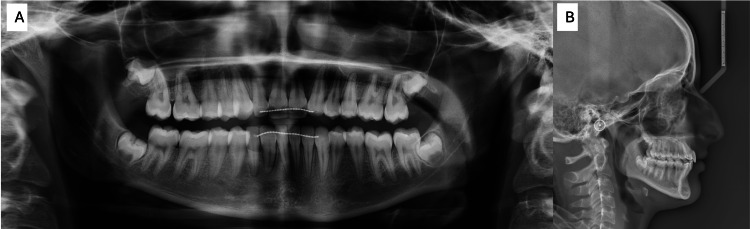
Post-treatment radiographs. The images show (A) post-treatment panoramic and (B) post-treatment lateral cephalogram.

Treatment was completed in 36 months. Maxillary space was closed successfully with esthetic canine reshaping. The mandibular transposition was aligned in situ without complications. The patient developed a mutually protected occlusion, yielding satisfactory esthetic results; however, periodontal compromise was still observed in relation to tooth #43 (Figures 5A-5C, 6A-6E, 7A, 7B).

**Figure 8:**
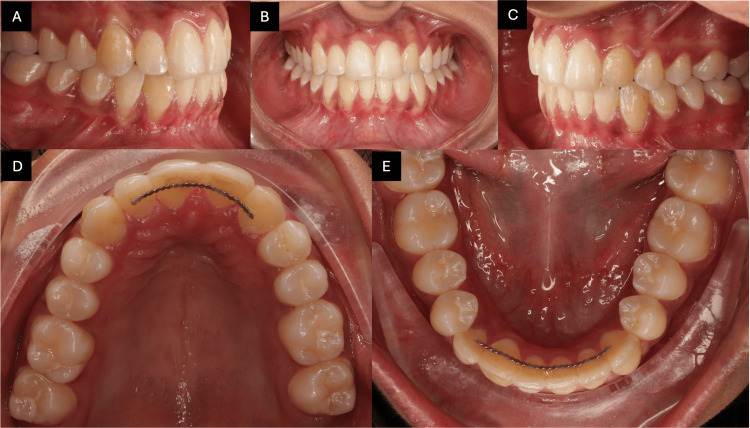
One-year follow-up intra-oral photographs. The images show (A) right buccal view, (B) frontal view, (C) left buccal view, (D) maxillary occlusal view, and (E) mandibular occlusal view.

At the one-year post-retention follow-up, intra-oral photographs demonstrated stable treatment outcomes with well-maintained alignment, proper occlusion, and no evidence of relapse tendency, thereby confirming the effectiveness of the chosen treatment and retention protocol (Figures 8A-8E).

## Discussion

Future considerations for such cases may include long-term monitoring of periodontal health, occlusal wear, and esthetic aging of reshaped teeth. Continued interdisciplinary collaboration remains essential in managing complex dental anomalies.

This patient benefited from timely intervention, accurate diagnosis, and an individualized treatment plan. The combination of canine substitution and acceptance of transposition allowed for efficient treatment without the need for extractions or surgical correction. Importantly, it preserved dental structures and periodontal health while delivering esthetic and functional results.

The decision to accept a complete mandibular lateral incisor-canine transposition rather than attempt correction was grounded in clinical and radiographic evidence. The roots were fully interchanged, and any attempt to reposition them would have posed a significant risk of root resorption or periodontal damage. Evidence in the literature indicates that, in such cases, alignment in the transposed position is a viable and often preferred alternative [4,6,8]. Moreover, careful torque control and crown positioning can ensure functional occlusion and acceptable esthetics with better gingival level. In this patient, the mandibular right canine (#43), which was transposed into the lateral incisor position, was torqued and reshaped occlusally to adapt harmoniously to the opposing maxillary canine. Together with the adjacent premolars, this created a group function occlusion on the right side.

From a functional standpoint, the conversion of a canine to a lateral incisor may compromise guidance during mandibular excursions. However, modern orthodontic biomechanics and occlusal adjustments can mitigate this risk by achieving a group function or mutually protected occlusion, depending on the individual case [9]. One of the main challenges in canine substitution cases is the functional adaptation of the maxillary first premolar. In this patient, the palatal cusp of the maxillary left first premolar (#24), which substituted for the canine, was selectively reduced (enameloplasty) and reshaped to eliminate potential working-side interferences. This modification allowed the premolar to contribute effectively to lateral guidance. Moreover, functional guidance was shared between the reshaped premolar (#24) and the maxillary anterior teeth, establishing a group function occlusion. Pure canine guidance could not be achieved, but the premolar was able to provide smooth lateral disclusion without interfering contacts.Regarding anterior guidance, the maxillary and mandibular incisors (including the reshaped #43 transposed into the lateral incisor position) maintained contact during protrusive movements, thereby providing effective anterior guidance.​​​​​​ This was critical to ensure posterior disclusion during forward mandibular excursions. Although canine guidance was not possible due to the substitution and transposition, the combination of incisors in protrusion and group function in laterotrusion provided a stable and functionally protective occlusal scheme. The final occlusion was characterized by anterior guidance in protrusive movement provided by the incisors, bilateral group function occlusion during lateral excursions achieved through reshaping of teeth #24 and #43, and the absence of non-working-side interferences.

The orthodontic management of maxillary lateral incisor agenesis has been the subject of extensive debate. While prosthetic replacement has its place in adult patients, canine substitution is often the preferred strategy in adolescents due to its conservative nature and potential to maintain alveolar bone integrity. Esthetic challenges posed by canine substitution, such as color mismatch, increased crown bulk, and gingival margin discrepancy, can be effectively managed with enameloplasty, composite resin buildups, and soft tissue recontouring. In this case, the reshaping of the canine and first premolar provided a pleasing esthetic result without the need for prosthetics or surgical intervention [6,10]. Therefore, this outcome highlights the importance of careful enameloplasty and occlusal reshaping in compensating for altered tooth positions and maintaining functional harmony.

During the initial clinical evaluation, gingival recession was observed on the labial aspect of tooth #43, the mandibular right canine. This recession was likely related to the initial transposed and ectopic eruption path of the tooth, which can contribute to alveolar bone dehiscence and thin labial tissue biotype [11,12]. Malpositioned teeth, especially in the canine region, are more susceptible to recession due to the prominence of the root and reduced surrounding bone.

Orthodontic alignment played a key role in improving the periodontal condition. By repositioning #43 within the alveolar housing, reducing its labial inclination, and improving its axial angulation, the tooth was brought into a more favorable position that supports better soft tissue adaptation [13]. The controlled orthodontic movement may have contributed to partial coronal migration of the gingival margin, improved cleansability, and reduced tension on the gingival tissue. Continued periodontal maintenance and care were carried out after debonding.

Clinical implications and limitations

Canine substitution represents a reliable and esthetic treatment option for managing lateral incisor agenesis in adolescents, eliminating the need for prosthetic replacement. Furthermore, in situations where a complete transposition is present and the roots are fully interchanged, accepting the transposed position may be the safer choice, as it avoids iatrogenic risks, such as root resorption or periodontal damage, while still allowing the achievement of functional occlusion and satisfactory esthetic outcomes. In this case, proper torque control and esthetic recontouring by enameloplasty were essential to simulate natural esthetics and achieve functional occlusion.

In terms of limitations, even with careful reshaping, the canine may not fully replicate the morphology, shade, or gingival margin position of a lateral incisor, which can result in a degree of esthetic compromise. Functionally, the establishment of group function may not completely substitute for ideal canine guidance in excursive movements. Moreover, in this case, canine substitution prevented correction of the class II molar relationship on the left side. Treatment planning is also case-dependent, as space availability, root angulation, and occlusal relationships may restrict the broader applicability of this approach. Finally, long-term follow-up is essential, as stability and periodontal health require ongoing evaluation and maintenance.

## Conclusions

This case demonstrates the successful orthodontic management of a rare and complex presentation involving unilateral maxillary lateral incisor agenesis and complete mandibular canine-lateral incisor transposition. The strategy of canine substitution in the maxilla and acceptance of the mandibular transposition provided a balanced, esthetic, and functional outcome without the need for extractions, implants, or prosthetic replacements. The long-term success of such cases depends on accurate diagnosis, interdisciplinary input, conservative biomechanics, and vigilant periodontal follow-up. This case adds to the clinical evidence supporting the viability of canine substitution and transposition acceptance as predictable solutions for complex anomalies in young patients.
